# Study on surface residual stress of 42CrMo steel treated by ultrasonic rolling extrusion

**DOI:** 10.1038/s41598-023-34203-x

**Published:** 2023-04-28

**Authors:** Haojie Wang, Xiaoqiang Wang, Yingjian Tian, Yuanfei Ling

**Affiliations:** School of Mechatronics Engineering, Henan University of Science and Technology, Luoyang, 471003 China

**Keywords:** Mechanical engineering, Scientific data

## Abstract

To obtain the distribution law of residual stress of 42CrMo steel processed by ultrasonic rolling extrusion process (UREP), according to the characteristics of the material treated by ultrasonic rolling extrusion process, 42CrMo steel quasi-static experiment and dynamic experiment are carried out, the improved Johnson–Cook model was proposed. Based on the 42CrMo constitutive equation, the ultrasonic rolling extrusion process finite element method and experiment of 42CrMo steel were carried out. The results show that the simulation value and experimental value of the residual stress distribution after ultrasonic rolling extrusion process of 42CrMo steel have good consistency, with the increase of static pressure and amplitude, the residual stress increases, and with the increase of rotational speed and feed speed, the residual stress decreases, a more accurate residual stress distribution law of ultrasonic rolling extrusion of 42CrMo steel is obtained.

## Introduction

As an ultra-high-strength steel, 42CrMo is widely used in the manufacture of wind power bearing rings due to its good strength, toughness and hardenability^1–3^. However, due to the fact that the wind power bearing rings are in a harsh working environment for a long time, the surface is prone to fatigue damage, resulting in wear, cracks and fractures. To improve the surface performance of parts and prolong the service life, surface strengthening technology is usually used to improve the surface properties of 42CrMo steel and improve the fatigue life of parts.

Conventional part surface strengthening methods include shot peening^4^, roll extrusion^5^, laser cladding^6^, polishing^7^, and EDM^8^. The shot peening processing efficiency is too low, the surface of the parts after laser cladding processing is uneven, for wind power bearing rings, the shape of the parts is curved, and the processing is not very convenient. EDM units are complex. Therefore, the ultrasonic rolling extrusion process (UREP) is proposed, which has a simple structure and easy operation, and will not cause strains of the surface structure after processing. UREP is based on the conventional extrusion method, combined with the rolling of the ball and ultrasonic vibration, so that the bearing surface produces severe plastic deformation, thereby generating residual compressive stress and work hardening layer on its surface, which is the key to improving the mechanical properties of the conventional surface strengthening method.

The generation of residual compressive stress can inhibit the formation of surface microcracks, which effectively improve the surface mechanical properties of parts, improve fatigue resistance and extend service life. Shen et al.^9^ studied the residual stress variation law of C-250 martensitic failure steel after grinding processing, and obtained the residual stress distribution on the surface and subsurface of the part. Wu et al.^10^ used the finite element method to analyze the residual compressive stress distribution of ultrasonic surface rolling process (USRP) single-point impact of 40Cr steel, and found that when the amplitude was 12 μm and the static pressure was 600 N, the residual compressive stress was the largest. Wang et al.^11^ studied the surface integrity of USRP of M50 bearing steel, which showed that the factors affecting the residual stress were feed speed, static pressure, and rolling time. Qin et al.^12^ studied the fatigue resistance of USRP of EA4T axle steel, and the bending fatigue strength of the workpiece surface after USRP processing increased by 46.9%, which he attributed to the improvement of residual compressive stress. Dang et al.^13^ studied the wear resistance of 300 M steel treated by USRP, which showed that the generation of residual compressive stress increased the hardness of parts by 30.9% and enhanced the wear resistance of 300 M steel. He et al.^14^ conducted finite element analysis on 18CrNiMo7-6 steel treated by single-pass USRP, and the results showed that when the radius of the tool head is small and the static pressure increases, the depth of the residual stress layer can be increased. Fan et al.^15^ studied the fretting fatigue behavior of ultrasonic surface rolling Ti-6Al-4 V alloy, and the results showed that the residual compressive stress generated by Ti-6Al-4 V alloy after ultrasonic surface rolling can inhibit the generation of microcracks and improve the crack growth life.

Zheng et al.^16^ established the residual stress analysis model of 7075 aluminum alloy USRP, with the increase of amplitude, roller radius and static pressure, the residual stress increased, which the calculated value of the analytical model was consistent with the experimental results. Luo et al.^17^ found that the depth of the plastic deformation layer of USRP-treated Ti-6Al-4 V alloys was 304.38 μm, and the increase of residual stress was caused by grain refinement and dislocation strengthening. Yang et al.^18^ found that excessive USRP will cause the internal grain size of GH4169 superalloy to increase from 58.23 to 280.7 nm, which reduces the residual stress and the wear resistance of GH4169 superalloy. Gong et al.^19^ found that when USRP times of Cr12MoV steel increased, the thickness of the plastic deformation layer increased significantly, which could inhibit the occurrence of microcracks. John et al.^20^ found that USRP can strengthen various ferrous and non-ferrous metals, induce nano-gradient layers, and improve the surface integrity of materials. Chen et al.^21^ found that the uranium treated by USRP increased the microhardness and residual stress, and the friction coefficient was reduced to 0.66 under the wear test, and the amount of wear was also greatly reduced. Peng et al.^22^ found that the surface integrity of USRP-treated 45 steel significantly affected the following degrees: USRP times, feed speed, static pressure, and amplitude. Xu et al.^23^ used the USRP method to improve the low-temperature mechanical properties of FH36 marine steel, and the results showed that the FH36 marine steel treated by USRP formed a residual stress layer of 2.6 mm, which improved the service life in low temperature environment.

Therefore, Scholars have conducted a lot of research on the residual stress of UREP and obtained the distribution regular of the residual stress of UREP, but rarely consider the improvement constitutive model of material, and analyse UREP simulation and experiment at the same time. This paper establishes the constitutive model of 42CrMo steel suitable for UREP, and the simulation analysis of UREP are carried out by finite element simulation ABAQUS software and simultaneously UREP experiments are conducted, which have good applicability to the surface properties of 42CrMo steel. The quasi-static experiment and dynamic experiment of 42CrMo steel are carried out, various stress–strain parameters of 42CrMo steel are obtained, the improved Johnson–Cook model is proposed by experimental results for the characteristics of UREP, it is very unreasonable to ignore the temperature softening effect caused by the adiabatic temperature rise for the conventional Johnson–Cook model. To describe the material properties of 42CrMo steel more accurately, on the basis of the conventional Johnson–Cook constitutive model, the adiabatic temperature rise softening term is added innovatively. The dynamic mechanical properties of 42CrMo steel can be better described, which makes UREP simulation test of 42CrMo steel more accurate. The improved Johnson–Cook constitutive model of 42CrMo steel can be well applied to the simulation process of UREP so that it can obtain the residual stress distribution of 42CrMo steel treated by UREP. The consistency of the UREP simulation results and experimental results verifies the accuracy of the improved Johnson–Cook constitutive model, which the distribution law of the residual stress of UREP can be well explored.

## Materials and methods

### Establishment of constitutive model of 42CrMo steel

**Figure 1:**
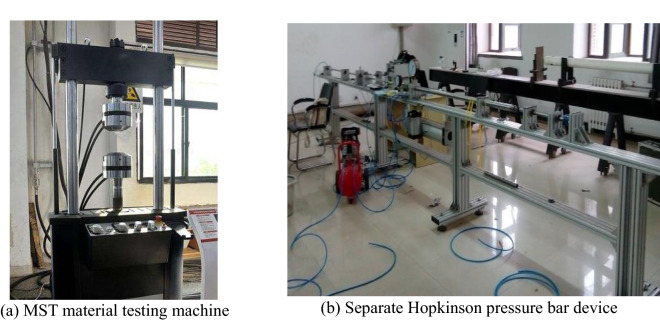
42CrMo quasi-static test.

The test piece is 42CrMo steel bar after quenching and grinding. The size of the test piece is Φ50 mm × 300 mm, and the hardness is 630 HV. The test piece is shown in Fig. 1. The main chemical composition is shown in Table 1. The physical parameters^24^ are shown in Table 2. The content of manganese and chromium in the material are more, which manganese can significantly improve the strength of the material due to the refinement of martensite, and the increase of chromium content can improve the hardenability.

**Table 1:** Chemical composition of 42CrMo steel.

Category	Element name						
	C	Mn	Cr	Mo	Si	Ni	Fe
42CrMo	0.37%	0.77%	0.98%	0.21%	0.15%	0.04%	97.44%

**Table 2:** Physical parameters of 42CrMo steel.

Category	Physical parameters				
	Density (kg/m^3^)	Elastic modulus (GPa)	Poisson ratio	Yield strength (MPa)	Thermal expansivity
42CrMo	7850	212	0.28	930	1.24 × 10^–5^

42CrMo steel quasi-static test selected MTS testing machine (as shown in Fig. 1a), the maximum axial load of the testing machine is 250 kN; The dynamic compression test is carried out by a separate Hopkinson compression rod device (Fig. 1b), which is mainly composed of an impact rod, an incident rod, a transmission rod and a specimen strip.

The quasi-static experiment is carried out at room temperature, the specimen is placed on the fixed platform of the experimental machine, the specimen is clamped by the fixed device, and the experimental machine is adjusted to press down at a constant speed. Strain gages are connected to both ends of the specimen to measure the actual strain value and convert the resulting engineering stress and engineering strain into true stress and true strain. The test parameters are shown in Table 3.

**Table 3:** 42CrMo steel physical parameters.

	Quasi-static (s^−1^)	Dynamic (s^−1^)
Strain rate	0.1 0.01 0.001	680 2500 3500 4800

The dynamic test is carried out at room temperature, the specimen is placed between the incident rod and the transmission rod, and the compression gun pressure and impact rod length are adjusted to control the strain rate. When the impact rod hits the incident rod, an elastic compression wave is generated in the incident rod and the impact rod, and due to the difference in wave impedance between the incident rod and the specimen, part of the wave is reflected in the form of a stretch wave, and the other part is transmitted to the transmission rod through the specimen. From the one-dimensional stress wave theory, the relationship between the engineering strain rate *ε*_1_ (*t*), the engineering *ε* (*t*) and the engineering stress *σ* (*t*) over time is shown in Eq. (1).

1 $$ \left\{ \begin{gathered} \sigma \left( t \right) = \frac{{A_{x} E}}{{A_{s} }}\varepsilon_{T} \left( t \right) \hfill \\ \varepsilon \left( t \right) = \frac{{2C_{0} }}{{L_{s} }}\int\limits_{0}^{t} {\varepsilon_{R} } \left( t \right)dt \hfill \\ \varepsilon_{1} \left( t \right) = \frac{{2C_{0} }}{{L_{s} }}\varepsilon_{R} \left( t \right) \hfill \\ \end{gathered} \right. $$

where *σ* (*t*) is the engineering stress of the specimen, *A*_*x*_ is the cross-sectional area of the pressure rod, *E* is the modulus of elasticity of the pressure rod, *A*_*s*_ is the cross-sectional area of the test piece, *ε*_T_ (*t*) is the amplitude of the transmitted wave, *ε* (*t*) is the engineering strain, *C*_0_ is the elastic wave velocity of the pressure rod, *L*_*s*_ is the total length of the specimen, *ε*_*R*_ (*t*) is the amplitude of the reflected wave,* ε*_1_ (*t*) is the engineering strain rate.

The cross-sectional area of the specimen material changes dynamically during the test, so the true stress strain of the material should be the ratio of the applied load to the instantaneous cross-sectional area. Therefore, the following formula is used to transform the engineering stress–strain curve in the result, and its calculation formula is shown in Eq. (2).

2 $$ \left\{ \begin{gathered} \sigma_{2} \left( t \right) = \sigma \left( t \right)\left[ {1 - \varepsilon \left( t \right)} \right] \hfill \\ \varepsilon_{2} \left( t \right) = - \ln \left[ {1 - \varepsilon \left( t \right)} \right] \hfill \\ w\left( t \right) = {{\varepsilon_{1} \left( t \right)} \mathord{\left/ {\vphantom {{\varepsilon_{1} \left( t \right)} {\left[ {1 - \varepsilon \left( t \right)} \right]}}} \right. \kern-0pt} {\left[ {1 - \varepsilon \left( t \right)} \right]}} \hfill \\ \end{gathered} \right. $$

where *σ*_2_ (*t*) is the true stress,* σ* (*t*) is the engineering stress of the specimen, *ε* (*t*) is the engineering strain, *ε*_2_ (*t*) is the true strain, *w* (*t*) is the true strain rate, *ε*_1_ (*t*) is the engineering strain rate.

**Figure 2:**
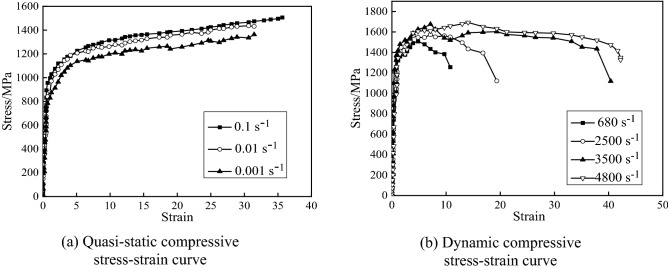
42CrMo quasi-static test.

It can be seen from Fig. 2a that there is a linear change between the stress and the strain value in the elastic stage, and it is found that 42CrMo steel has no obvious yield platform in the compression process, but with the increase of the strain rate, the yield strength is improved, and when the strain value is about 0.005, the yield stress of the material is about 900 MPa. Then, in the strain hardening stage, the stress increase amplitude is not obvious with the increase of the strain variable, and the slope of the stress–strain curve under each strain rate at this stage remains almost unchanged, indicating that the strain hardening rate does not change much at this stage.

It can be seen from Fig. 2b that the stress–strain curve also changes linearly in the elastic phase, and the elastic modulus of the material increases significantly compared with the low strain rate in the high strain rate range. In the yield phase, the dynamic compression process has a significant yield plateau, and the yield strength increases significantly as the strain rate increases. It can be seen that the static mechanical properties and dynamic properties of 42CrMo steel are significantly different, that is, the yield strength is increased at high strain rates.

According to the characteristics of the body-centered cubic metal belonging to 42CrMo, based on the results of quasi-static and dynamic tests, the J–C constitutive model is proposed to describe its behavior characteristics, and the J–C model is improved accordingly according to the ultrasonic rolling strengthening characteristics. In the ultrasonic rolling intensification process, the overall heat production is not large, and the heat balance state is ideal when the coolant supply is sufficient, and the overall temperature of the contact area is basically stable at a level slightly higher than room temperature. However, due to the high strain rate, the local instantaneous heat production in the plastic deformation zone is large, and the instantaneous temperature rise at the position of severe plastic deformation has an inevitable influence on its strain characteristics. Kapoor et al.^25^ studied the problem of high strain rate deformation, and gave the calculation process of the local temperature rise of the deformation zone as shown in Eqs. (3) and (4).

3 $$ \Delta Q \approx \eta \cdot \Delta W $$

4 $$ \Delta T(\varepsilon ) = \frac{\eta }{{\rho \cdot C_{v} }} \cdot \int_{0}^{\varepsilon } {\sigma_{e} d} \varepsilon $$

where *△Q* is heat production, *η* is the power heat conversion coefficient, *△W* is plastic deformation work, *△T* (*ε*) is a local temperature rise, *ρ* is the density of the material, *C*_*v*_ is temperature-dependent specific heat, *σ*_*e*_ is stress, *ε* is strain.

In the conventional J–C model, the influence of the adiabatic temperature rise of the material at high strain rates is not taken into account. Through the quasi-static and dynamic tests of 42CrMo steel, the stress–strain curve of 42CrMo steel at the strain rate from 0.001 to 4800 s^−1^ was obtained, the results show that the mechanical properties of 42CrMo steel at high strain rate are completely different from those at the conventional static compression rate, which is caused by the thermal softening effect. The thermal softening effect means that the heat generated by the plastic deformation of 42CrMo steel under high strain rate cannot be quickly diffused to the outside environment, and the accumulation leads to the rise of the material temperature, the thermal softening effect has a great impact on the mechanical properties of the material. It is known that the density of 42CrMo steel is 7850 kg/m^3^, the work-heat conversion coefficient is 0.9, and the local temperature rise is 18 °C when the quasi-static strain rate is 680 s^−1^ and 150 °C when the dynamic strain rate is 4800 s^-1^ in the stress–strain curve. Obviously, it is very unreasonable to ignore the temperature softening effect caused by the adiabatic temperature rise Therefore, in the improved J–C constitutive model, the adiabatic temperature rise softening term is added to replace the difference between the overall temperature and the ambient temperature. Since UREP is processing at room temperature, the effect of the difference between the test temperature and the room temperature can be ignored. But the adiabatic temperature rise is independent and must be considered separately, the improved J–C model is corresponded to the facts. Therefore, the improved J–C constitutive model can better describe the dynamic mechanical properties of 42CrMo steel on impact and make UREP simulation test of 42CrMo steel more accurate. The introduction of thermal softening term caused by adiabatic temperature rise in the J-C constitutive equation is the key to improving the J–C model and the conventional J–C model. Therefore, the temperature rise effect term can be added when applied to the ultrasonic rolling strengthening problem, and the J–C model can be improved as shown in Eq. (5).

5 $$ \sigma = \left( {A + B\varepsilon^{n} } \right)\left( {1 + C\ln \dot{\varepsilon }^{\prime}} \right)\left( {1 - T^{*m} } \right) $$

In the improved model, where $\sigma$ is the effective yield stress, $A$ is the initial yield stress, $B$ is the strain hardening coefficient, $\varepsilon$ is the plastic strain, $n$ is the hardening exponent, $C$ is the strain rate strengthening coefficient, $\dot{\varepsilon }^{\prime}$ is the normalized effective plastic strain rate, $T^{*}$ is the homologous temperature, $m$ is the thermal softening exponent of the material. The normalized effective plastic strain rate is shown Eq. (6).

6 $$ \dot{\varepsilon }^{\prime} = \frac{{\dot{\varepsilon }}}{{\dot{\varepsilon }_{0} }} $$

where $\dot{\varepsilon }^{\prime}$ is the normalized effective plastic strain rate, $\dot{\varepsilon }$ is the plastic strain rate, $\dot{\varepsilon }_{0}$ is the reference strain rate. The homologous temperature is shown Eq. (7).

7 $$ T^{*} = \frac{\Delta T}{{T_{m} - T_{r} }} $$

where $T^{*}$ is the homologous temperature, $\Delta T$ is the local temperature rise term, $T_{m}$ is the melting point temperature, $T_{r}$ is reference temperature, which can also be taken as room temperature. Compared with the conventional J–C model, the local temperature rise term $\Delta T$ is used to replace the term of the difference between the overall temperature and the room temperature. Improved J–C model parameters of 42CrMo steel is shown in Table 4. The constitutive model of 42CrMo material suitable for UREP can be obtained as shown in Eq. (8). The improved J–C model will provide an accurate constitutive model for UREP simulations.

8 $$ \sigma = \left( {1059 + 528\varepsilon^{0.22} } \right) \cdot \left( {1 + 0.008\ln \dot{\varepsilon }^{\prime}} \right) \cdot \left( {1 - T^{*m} } \right) $$

where $\sigma$ is the effective yield stress, $\varepsilon$ is the plastic strain, $\dot{\varepsilon }^{\prime}$ is the normalized effective plastic strain rate, $T^{*}$ is the homologous temperature, $m$ is the thermal softening exponent of the material.

**Table 4:** Improved J–C model parameters of 42CrMo steel.

*A* (MPa)	*B* (MPa)	*C*	*n*	*m*	Melting temperature (K)	Transition temperature (K)
1059	528	0.008	0.211	1	1668	20

### UREP experiment

**Figure 3:**
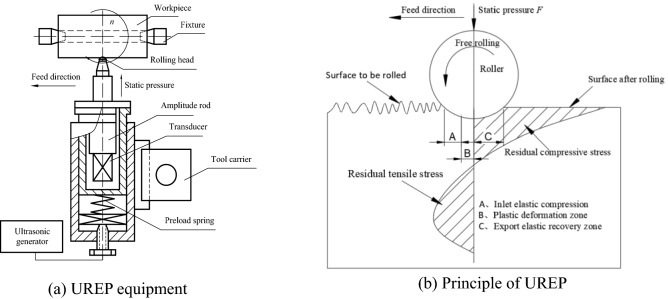
UREP machining schematic.

As a new plastic forming technology, ultrasonic rolling extrusion process (UREP) is ultrasonic processing and roller extrusion processing integration of composite processing technology. As shown in Fig. 3a, UREP equipment is composed of ultrasonic generator, transducer, amplitude rod, rolling head. The ultrasonic generator emits a high-frequency oscillating electrical signal, and the function of the transducer is to turn the electrical signal into mechanical vibration, and the high-frequency mechanical vibration is amplified and transmitted to the high-strength rolling head through the amplitude rod. The static pressure applied by the spring acts on the rolling head, so that the rolling head is close to the surface of the specimen. The principle of UREP is shown in Fig. 3b, Under the combination of static pressure and high-frequency mechanical vibration, UREP tool head produces violent plastic deformation in the specimen, the internal elastic deformation zone (A section) recovers deformation, and the external plastic deformation zone (B section) prevents its deformation, thereby generating residual compressive stress and elastic recovery zone on its surface (C section), thereby improving the surface performance of 42CrMo steel. During UREP, it produces "peak load shifting", which refers to flatten the bulge and fill in the recessed area so that the surface of the workpiece produces better quality.

**Figure 4:**
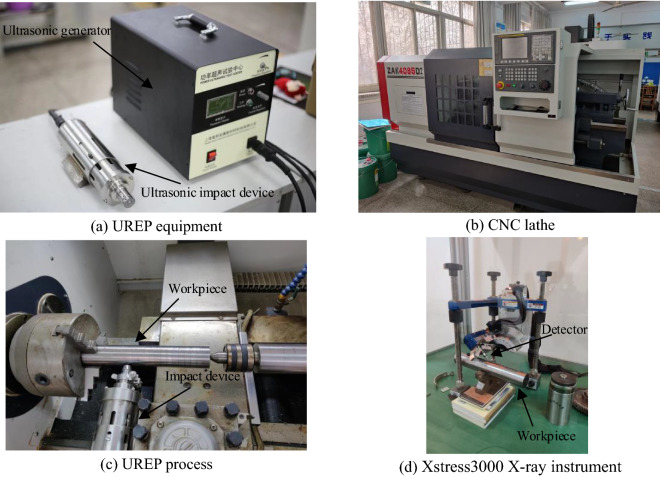
UREP machining process.

UREP equipment is composed of ultrasonic generator, transducer, amplitude rod and rolling head, as shown in Fig. 4a. UREP test is carried out on ZAK4085D1 CNC lathe, as shown in Fig. 4b. The static pressure is based on the scale of the luffing rod, where the compression of 1 mm represents the static pressure of 50 N. The processing length of each group of cylindrical bars is 20 mm. Using UREP equipment to process 42CrMo steel is shown in Fig. 4c. The residual stress (*RS*) of the surface layer is measured by the Xstress3000 X-ray stress instrument, as shown in Fig. 4d. The test selects the test voltage of 30 kV, the current of 7 mA and the Cu target *K*_*α*_ ray to irradiate the surface of the test piece, the radiation area is 1 mm^2^, and the sin*2φ* method was selected to measure the surface residual stress. The value of the *φ* angle is 0°, 45°, and three points are taken on the same test piece to measure, and then the average value is obtained. To measure the residual stress in the depth direction more accurately and without introducing external stress, DM electrolytic polishing instrument is used to electropolish the specimen. The electrolytic cell is injected with saturated NaCl solution, the polishing voltage is set to 30 V, the polishing current is set to 1.5 A. The polishing head, cotton stained with electrolyte, is contact with the workpiece directly, and the detection area is uniformly electrolytically polished and stripped each time, which the polishing time determines the thickness of each polishing layer, the longer the polishing time, the deeper the thickness of the polishing layer. The thickness of each polished layer is measured by the micrometer whose the accuracy is 1 μm. Simultaneously, the residual stress of each layer of the workpiece is measured using the Xstress3000 X-ray stress instrument. Then the residual stress measurement steps are cycled sequentially, including electrolytic polishing, polishing layer thickness measurement, and residual stress detection, until the measurement of the residual stress at required position is completed completely in the depth direction.

### UREP finite element simulation

**Figure 5:**
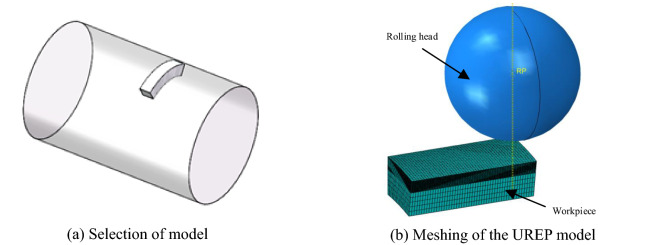
Establishment of UREP model.

UREP simulation were performed using the Explicit module in ABAQUS software. During UREP, the contact type between the 42CrMo steel and the rolling head is point contact, and the stress field is symmetrically distributed during ultrasonic vibration. The St. Venant principle^26^ explains that the load only affects the stress distribution near the applied area, while the farther regions are not affected. Therefore, the impact force generated by the rolling head in this simulation can be regarded as having an effect on only a small area. To fasten the simulation solution, this paper selects a 1/36 3 mm thick hollow cylinder for simulation analysis as shown in Fig. 5a, this thickness range is universal and referential for cylindrical workpieces. For cylindrical machined parts, the simulation solution time will be greatly extended by establishing a complete cylinder part model. However, for the UREP process, the influence of UREP on the surface performance of the material is mainly studied, and the thickness of the 3 mm thick part can meet the requirements for the study of the residual stress of the workpiece along the depth direction. However, this is only applicable to cylindrical workpieces with a diameter of 50 mm, when machining parts of other shapes or cylindrical workpieces of different diameters, the simulation model will not be applicable, and the simulation model needs to be rebuilt, and UREP model is shown in Fig. 5b.

To avoid "self-locking" and "hourglass" phenomena due to mesh distortion, the C3D8R which is selected for explicit on the element type was chosen to divide the mesh of the processed specimen. The mesh is refined on the surface of the test piece, the size is 0.05 mm, and in the inner area of the test piece, the grid with a size of 0.015 mm is normally divided, and the model is divided into a total of 57,750 meshes, as shown in Fig. 4d. Rolling head is a diameter of 10 mm high hardness tungsten steel ball, compared with the stiffness of 42CrMo specimen, tungsten steel ball stiffness is larger, to fasten the simulation process of the calculation time, improve the solution efficiency, the rolling head as an ideal rigid body, high hardness tungsten steel ball using analytical rigid body model. The analysis step is set to dynamic explicit, the analysis step time is set to 0.1 s, the contact type is set to surface-to-surface contact, the contact property is set to general contact, the normal behavior is set to hard contact, the friction formulation in tangential behavior is set to penalty, and the friction coefficient is set to 0.02. The center of the rolling ball is specified as the reference RP1, and the 1/36 3 mm thick hollow cylinder part center is designated as the reference point RP2, and the load and displacement boundary conditions are applied to the reference point. In the load module, the static pressure is applied to the reference point RP1 in the center of the rolling ball, pointing vertically to the surface of the part along the Y direction, where the static pressure is 200 N, 280 N, 350 N, 480 N, 600 N respectively. The actual UREP simulation process is that the rolling ball vibrates ultrasonically in the Y direction while rotating around the Z axis and feeding along the Z axis, and the cylindrical part only rotates along the Z axis. The setting of boundary conditions is that limit the translational degree of freedom of the rolling ball in the X direction, the rotational degree of freedom in the Z direction and Y direction, and limit the translational degree of freedom of the cylindrical parts in the X, Y and Z directions, at the same time, limit the rational degree of freedom in the X direction and Y direction of the cylindrical workpiece, and only allow rotation along the Z axis. Since the analysis step is set to dynamic explicit, the boundary conditions applied to both the rolling ball and the workpiece need to be specified amplitude. The boundary condition is set in velocity. According to the time of the analysis step, the specific values are converted the rotational speed, the feed rate, etc. The reference point RP1 of the rolling ball is set to feed in the Z axis while rotating around the Z axis, and the type of amplitude is set in tabular form. The ultrasonic vibration is reflected by the vertical vibration of RP1, the reference point of the rolling ball in the Y direction, where the type of amplitude is set in the form of periodic. The circular frequency is set in the amplitude module, and the vibration frequency is calculated to 125,664. The reference point RP2 of the cylindrical workpiece is set to rotate around the Z circumference for UREP simulation.

In UREP simulation processing, the force of the tool head on the surface of the workpiece is the superposition of radial ultrasonic high-frequency vibration and static pressure. Since static pressure and amplitude cannot be added to one analysis step in the Explicit module, the high-frequency vibration applied to the tool head is replaced by the static pressure itself, and the sine wave function is directly defined in the static pressure of the tool head. Its expression is shown in Eq. (9).

9 $$ F\left( t \right) = A_{0} + \sum\limits_{m = 1}^{\infty } {\left( {a_{n} \cos j\omega t + b_{n} \sin j\omega t} \right)} $$

where $F\left( t \right)$ is dynamic impact, $A_{0}$ is the inital amplitude, $a_{n}$ is the first simple harmonic amplitude, $j$ is the simple harmonic number, $\omega$ is the ultrasonic angular frequency, $t$ is the time of simple harmonic motion, $b_{n}$ is the second simple harmonic amplitude. Since the tool head movement is in the form of a sine wave, so $m = 1$, $a_{n} = 0$. The static pressure is converted into the initial amplitude to indirectly represent it, and the dynamic impact force and simple harmonic amplitude are solved. The expression as a function of the dynamic impact force is shown in Eq. (10).

10 $$ F\left( t \right) = A_{0} + b_{1} \left( {\sin \omega t} \right) $$

where $F\left( t \right)$ is the simplified dynamic impact, $A_{0}$ is the initial amplitude, $b_{1}$ is the simple harmonic amplitude, $\omega$ is the ultrasonic angular frequency, $t$ is the time of simple harmonic motion, The angular frequency of ultrasonic vibration is shown in Eq. (11).

11 $$ \omega = 2\pi f_{r} $$

where $\omega$ is the ultrasonic angular frequency, $f_{r}$ is the ultrasonic vibration frequency, $f_{r}$ is 20 kHz in UREP test.

### Orthogonal experimental design of UREP

An orthogonal experimental design is adopted for UREP. The processing parameters are workpiece speed *n*, feed speed *f*, amplitude *A* and static pressure *F*. A four factor and five level design L25(5^4^) is selected for the experiment. The factor level setting table is shown in Table 5.

**Table 5:** Factor level setting table.

Level	Process parameters of UREP			
	Speed (A)	Feed rate (B)	Amplitude (C)	Static pressure (D)
	*n* (r/min)	*f* (mm/min)	*A* (μm)	*F* (N)
1	130	15	5	200
2	250	25	10	280
3	340	30	15	350
4	490	40	20	480
5	630	55	25	600

## Results and discussion

The orthogonal experimental results are shown in Table 6.

**Table 6:** Orthogonal test results.

Test number	Horizontal combination	$\sigma$(MPa)
1	$A_{1} B_{1} C_{1} D_{1}$	− 901
2	$A_{1} B_{2} C_{2} D_{2}$	− 983
3	$A_{1} B_{3} C_{3} D_{3}$	− 1031
4	$A_{1} B_{4} C_{4} D_{4}$	− 1113
5	$A_{1} B_{5} C_{5} D_{5}$	− 1153
6	$A_{2} B_{1} C_{2} D_{3}$	− 1028
7	$A_{2} B_{2} C_{3} D_{4}$	− 1092
8	$A_{2} B_{3} C_{4} D_{5}$	− 1160
9	$A_{2} B_{4} C_{5} D_{1}$	− 940
10	$A_{2} B_{5} C_{1} D_{2}$	− 939
11	$A_{3} B_{1} C_{3} D_{5}$	− 1151
12	$A_{3} B_{2} C_{4} D_{1}$	− 937
13	$A_{3} B_{3} C_{5} D_{2}$	− 1012
14	$A_{3} B_{4} C_{1} D_{3}$	− 971
15	$A_{3} B_{5} C_{2} D_{4}$	− 1064
16	$A_{4} B_{1} C_{4} D_{2}$	− 997
17	$A_{4} B_{2} C_{5} D_{3}$	− 1053
18	$A_{4} B_{3} C_{1} D_{4}$	− 1038
19	$A_{4} B_{4} C_{2} D_{5}$	− 1116
20	$A_{4} B_{5} C_{3} D_{1}$	− 907
21	$A_{5} B_{1} C_{5} D_{4}$	− 1121
22	$A_{5} B_{2} C_{1} D_{5}$	− 1082
23	$A_{5} B_{3} C_{2} D_{1}$	− 898
24	$A_{5} B_{4} C_{3} D_{2}$	− 979
25	$A_{5} B_{5} C_{4} D_{3}$	− 1019

### Effect of rotational speed on residual stress layer depth

When the static pressure is 350 N, feed rate is 30 mm/min, the amplitude is 15 μm, UREP simulations and tests are carried out under different rotational speed. The 42CrMo steel cylindrical workpiece with the diameter of 50 mm is fixed on the horizontal ZAK4085D1 CNC lathe spindle, the ultrasonic impact equipment is fixed on the tool holder of the CNC lathe. The front end of the ultrasonic impact equipment is a rotatable carbide ball with a diameter of 10 mm. Set the same feed speed in the FANUC numerical control system, the fixed amplitude of the ultrasonic generator is 15 μm. According to the different sizes of the spindle speed, set different spindle speeds in the FANUC system to determine the processing parameters. Meanwhile, adjust the compression amount of the tail spring of the ultrasonic impact equipment to determine the static pressure, 5 UREP experiments are carry out. Use lubricating oil for cooling during UREP. According to the processing length of each section, the processed workpiece is cut on the Wire cut Electrical Discharge Machining (WEDM), and the part inspection area is wiped clean with alcohol cotton. Then 400, 800, 1000, 1500, and 2000 grit paper are used to polish the cross-section of the specimen, DM electrolytic polishing instrument was used to electrolytically polish the specimen, the electrolytic cell was injected with saturated NaCl solution, the polishing voltage is set to 30 V, the polishing current is set to 1.5 A. The polishing head, cotton stained with electrolyte, is contact with the workpiece directly, and the detection area was uniformly electrolytically polished and stripped each time. The thickness of the polished layer is measured by the micrometer screw, whose the accuracy is 1 μm. When the thickness of the polished layer is measured, the residual stress of the surface layer of the specimen is determined by Xstress3000 X-ray stress instrument, which the test voltage is 30 kV, the current is 7 mA, the copper target *Kα* ray is selected to irradiate the surface of the specimen, the radiation area is 1 mm^2^. The residual stress of the surface is measured by sin2*φ* method, the value of the *φ* angle is 0° and 45°, and three points are measured on the same specimen area to find its average value. Then the residual stress measurement steps are cycled sequentially, including electrolytic polishing, polishing layer thickness measurement, and residual stress detection.

**Figure 6:**
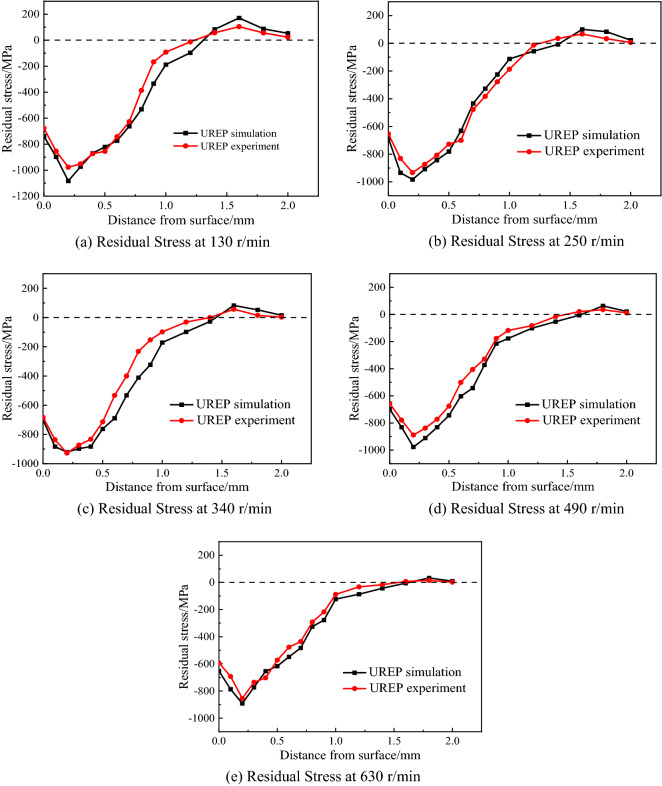
Residual stress of UREP at different rotational speed.

Figure 6 shows that the trend of change between the experimental value and the simulated value is roughly the same. UREP simulated value is greater than the experimental value. The overall trend is the "spoon shape". But the trend is very sharp. This may be due to a large selection range of speed ranges. With the increase of rotational speed, the maximum residual stress on the surface of the workpiece after UREP treatment becomes smaller and smaller. When the rotational speed is 130 r/min, the residual stress on the surface of the workpiece after UREP treatment is the largest. The residual stress of the surface of the workpiece after UREP treatment is the residual compressive stress, with the layer depth increases, the residual stress gradually increases, reaching a maximum value of 1083 MPa at a layer depth of 0.2 mm, then gradually decreases until it slowly becomes positive, and then approaches around 0. With the rotational speed increases, the residual compressive stress gradually decreases. When the rotational speed is low, the uniformity of the processing of the workpiece in the circumferential direction can form a regular-shaped work hardening layer on the surface of the specimen, that is, the same degree of plastic deformation, so that the degree of plastic deformation increases, thereby significantly increasing the residual compressive stress. When the speed increases, the residual compressive stress decreases significantly. This is because the degree of plastic deformation of the specimen in the circumferential direction is weakened per unit time, which reduces the residual compressive stress. Similarly, 42CrMo steel cylindrical workpiece rotate at high speed driven by the horizontal CNC lathe chuck, when the workpiece in the circumferential direction processed by the rolling ball, it will appear in some areas that are not treated by UREP, which causes the inadequate process of ultrasonic rolling extrusion. In other words, insufficient rolling extrusion leads to the work hardening layer formed unevenly, when the same area is not fully rolled, the rolling ball feeds along the axial direction under the drive of the horizontal CNC lathe tool holder, the unmachined area is ignored. Therefore, whether it is seen from the circumferential direction or axial direction, in terms of the 42CrMo steel cylindrical workpiece treated by UREP, too large speed will make the processing area discontinuous and uneven, resulting in reduced processing stability and uniformity at the same time, which produces the skip zone on the surface of the workpiece and the work hardening layer with the ununiform degree of deformation, finally, it will prompt the compressive residual stress to decrease.

### Effect of feed rate on residual stress layer depth

**Figure 7:**
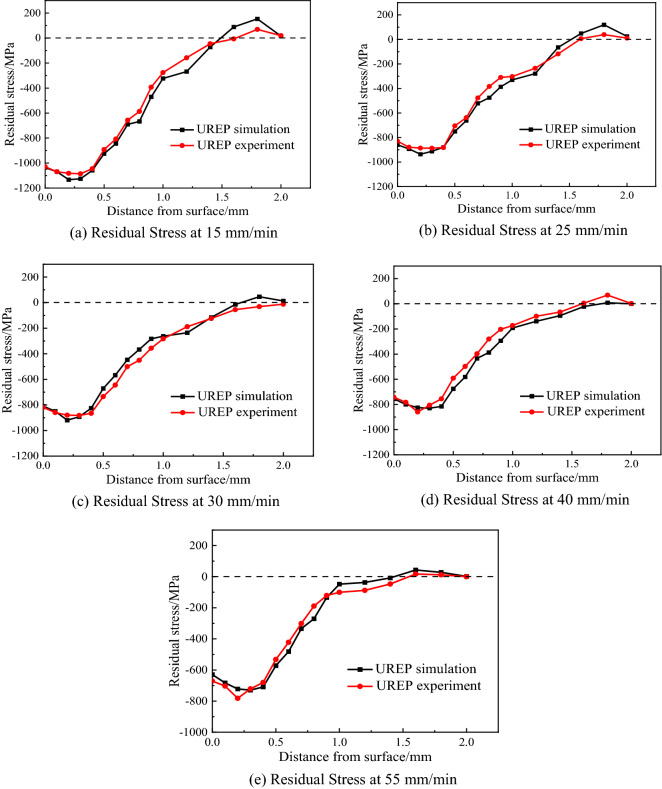
Residual stress of UREP at different feed rate.

When the rotational speed is 340 r/min, static pressure is 350 N, the amplitude is 15 μm, UREP simulations and tests are carried out under different feed rate. Similarly, the same speed is entered in the FANUC numerical control system of ZAK4085D1 CNC lathe, the ultrasonic generator is set to fix amplitude of 15 μm, which the static pressure is determined by the compression of the ultrasonic impact equipment tail spring. The 5 UREP tests are performed, and the process is cooled by oil. According to the processing length of each section, the processed workpiece is cut on the WEDM, and measure the thickness of the polished layer with the micrometer screw. Meanwhile, use Xstress3000 X-ray stress instrument to measure the residual stress on the surface of the polished workpiece, and cycle the electrolytic polishing, polishing layer thickness measurement, residual stress detection and other steps in turn. Figure 7 shows that the trend of change between the experimental value and the simulated value is roughly the same. Compared with Fig. 6, Fig. 7 shows that the residual stress trend in is relatively flat. This shows that the influence of feed speed on the residual stress is less than the rotational speed, which will lead to generate a relatively gentle process of reducing and increasing the residual stress. With the increase of feed rate, the maximum residual stress on the surface of the workpiece after UREP treatment becomes lower and lower. Similarly, the max residual compressive stress is located at a depth of 0.2 mm. When the feed rate is 15 mm/min, the simulated valued of max residual compressive stress is − 1132.843 MPa, while experimental valued is − 1082.637 MPa. Since several lengths are machined on a bar stock, it needs to be cut open for measurement, resulting in the release of residual stress, and the experimental value of residual stress will be smaller than the simulated value. The effect of feed rate on residual stress is consistent with the rotational speed, with the feed rate increases, the residual stress also decreases. This is because when the feed rate increases, when the rolling ball has not fully rolled the unmachined area, the rolling ball moves in the axial direction under the fast feed rate so that the rolling of the processing area is adequate and the continuous work-hardening layer cannot be formed. The machining continuity of the axial surface of the 42CrMo specimen is weakened, and the unmachined area will appear, resulting in a weakening of the degree of plastic deformation and a decrease in the residual compressive stress. When the feed speed decreases, the processing continuity of the specimen in the axial direction is enhanced, and each part along the axial direction is fully plastically deformed, so that the residual compressive stress increases significantly.

### Effect of static pressure on residual stress layer depth

**Figure 8:**
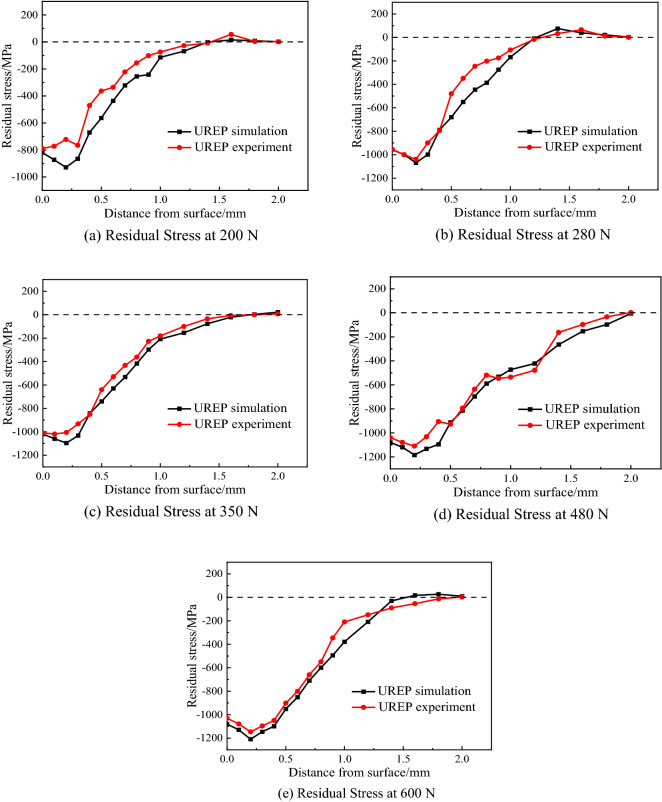
Residual stress of UREP at different static pressure.

When the rotational speed is 340 r/min, feed rate is 30 mm/min, the amplitude is 15 μm, UREP simulations and tests are carried out under different static pressures. It is set to be the same speed and feed speed on the FANUC numerical system of ZAK4085D1 CNC lathe and fixed the amplitude of 15 μm by the ultrasonic generator. According to the different sizes of the static pressure value, adjust the different compression amounts of the tail spring of the ultrasonic impact equipment to obtain different static pressures. 5 UREP tests are conducted. According to the processing length of each section, the specimen is cut on the WEDM and electrolytically polished by DM electrolytic polishing instrument. The thickness of the polished layer is measured by the micrometer. The residual stress on the surface of the polished workpiece is measured by the Xstress3000 X-ray stress instrument, and the step of measurement is taken circularly. Figure 8 shows that the trend of change between the experimental value and the simulated value is roughly the same. The change curve of residual stress with static pressure is also the "spoon shape". With the increase of static pressure, the maximum residual stress on the surface of the workpiece after UREP treatment also increase. The max residual compressive stress is located at a depth of 0.2 mm. When the static pressure is 600 N, the simulated valued of max residual compressive stress is − 1209.746 MPa, while experimental valued is − 1146.325 MPa. When the layer depth increases to 2 mm, the residual stress the surface of the workpiece after UREP treatment tends to be around 0. Compared with rotational speed and feed rate, the effect of static pressure to residual stress is severe. The influence of static pressure on residual stress tends to be steep. This shows that static pressure is the process parameter that has the greatest influence on residual stress on the surface of the workpiece after UREP treatment. As the layer depth increases, the residual stress gradually increases to a maximum value and then decreases, and gradually approaches 0 at a layer depth of 2 mm. This is because when the static pressure increases, the degree of plastic deformation on the surface of the specimen after UREP treatment also increases, so that the residual compressive stress increases significantly. Under the effect of ultrasonic softening, the degree of surface grain refinement and the grain refinement layer deepens also increases, resulting in an increase in residual compressive stress. Based on the principle of UREP, the previous process of UREP is precision turning, which leaves residual tensile stress on the surface of the cylindrical workpiece. When the cylindrical workpiece is treated by UREP, the residual tensile stress on the surface is converted into residual compressive stress. With the increase of layer depth, the residual compressive stress gradually increases to the maximum, and then decreases to 0. UREP refines the grains on the surface of the workpiece, which causes the grains to displace and slip at the same time. It will make it difficult to be extruded and deformed for the workpiece treated by UREP. Therefore, this improves the strength and hardness of the surface of the workpiece, which greatly improves the fatigue life of the workpiece.

### Effect of amplitude on residual stress layer depth

**Figure 9:**
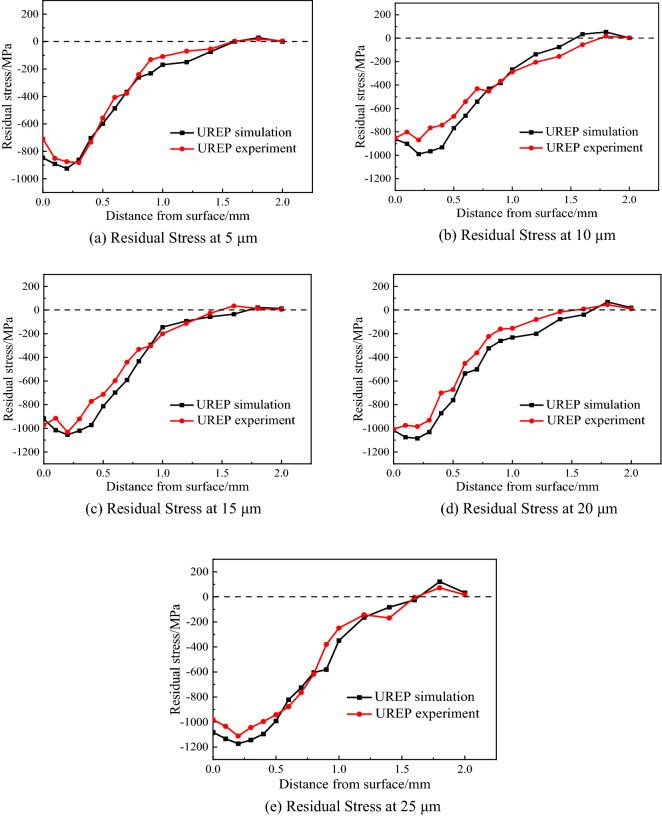
Residual stress of UREP at different amplitude.

When the rotational speed is 340 r/min, feed rate is 30 mm/min, the feed rate is 30 mm/min, UREP simulations and tests are carried out under different static pressures. By FANUC numerical system of ZAK4085D1 CNC lathe, set the same speed, feed speed, fix the vibration amplitude of the ultrasonic impact equipment, adjust the vibration amplitude on the ultrasonic generator to obtain different amplitudes. 5 UREP experiments are carried out. The treatment of the specimen and measurement of residual stress are as same as sections "Effect of rotational speed on residual stress layer depth", “Effect of feed rate on residual stress layer depth”, and "Effect of static pressure on residual stress layer depth". Figure 9 shows that the trend of change between the experimental value and the simulated value is roughly the same. With the increase of amplitude, the maximum residual stress on the surface of the workpiece after UREP treatment also increase. UREP experimental value is lower than the simulated value when the amplitude is 25 μm, the simulated valued of max residual compressive stress is − 1172.936 MPa, while experimental valued is − 1111.325 MPa. When the layer depth is between 0.2 and 1 mm, the residual stress decreases rapidly. When the layer depth increases to 2 mm, the residual stress the surface of the workpiece after UREP treatment tends to be around 0. The effect of amplitude on the workpiece is the same as the effect of static pressure on the workpiece. This is because when the amplitude of the tool head is increased, the impact of the tool head on the workpiece surface can be increased. The energy transmitted by the ultrasonic power to the workpiece surface can be enhanced, so that the plastic deformation degree of surface of the workpiece is intensified. Thereby generating a strong work-hardening layer on the surface of the workpiece, so that the residual compressive stress on the surface of the workpiece is significantly increased. Larger amplitude will produce greater energy to the workpiece, which can quickly improve the tool marks and residual tensile stress left by the previous process turning. Combined the action of static pressure with the high-frequency vibration hammering, the tool marks will be flattened, resulting in the "peak load shifting", the residual tensile stress is converted into residual compressive stress at the same time, which greatly inhibits the occurrence of cracks. The increase in amplitude is actually the improvement of the hammering speed when the rolling ball processes the workpiece on its surface, the higher the amplitude, the greater the impact force of the rolling ball hammering the surface of the workpiece. Combined static pressure with high-frequency hammering, the surface of the workpiece produces severe plastic deformation, forming the work hardening layer, thereby improving the fatigue resistance of the workpiece. UREP technology can effectively replace conventional grinding, which prompts greatly production efficiency and improves the performance of workpiece so that the processing cost of the workpiece does not increase at the same time, improving the surface performance of workpiece.

From the previous analysis, it can be concluded that static pressure and amplitude have a greater influence on the residual stress on the surface of the workpiece treated by UREP, while the effect of rotational speed and feed rate on the residual stress is small, so if increasing the surface performance of the material and improve the fatigue life, it should increase the static pressure and amplitude in the process parameters as much as possible.

## Conclusions

In this paper, based on the improved Johnson–Cook model, ABAQUS software is used to carry out 42CrMo steel of ultrasonic rolling extrusion process finite element simulation experiment, simultaneously, 42CrMo ultrasonic rolling extrusion process experiment is carried out to verify the accuracy of the simulation model. The following conclusions are obtained:

1. Through the quasi-static experiment and dynamic experiment of 42CrMo steel, the parameters in the improved Johnson–Cook model can be well used in the 42CrMo ultrasonic rolling extrusion process finite element simulation model.
2. The residual stress measured by the XStress3000 X-ray stress instrument is approximate to the simulated value. The simulated values obtained are well consistent with the experimental values. The established simulation model can be used to simulate the actual working conditions of 42CrMo ultrasonic rolling extrusion process, which provides an effective method for the design of USRP parameters.
3. The distribution law of residual stress of 42CrMo steel is obtained. The finite element simulation results and experimental results show that the residual stress gradually increases with the increase of the rotational speed, and the static pressure increases. As the feed rate increases, the residual stress gradually decreases. With the increase of static pressure, the residual stress gradually increases, and with the increase of amplitude, the residual stress gradually increases.

## Data Availability

The data that support the findings of this study are available on request from the corresponding author. The data are not publicly available due to privacy or ethical restrictions.
